# The hop constituent xanthohumol exhibits hepatoprotective effects and inhibits the activation of hepatic stellate cells at different levels

**DOI:** 10.3389/fphys.2015.00140

**Published:** 2015-05-06

**Authors:** Ralf Weiskirchen, Abdo Mahli, Sabine Weiskirchen, Claus Hellerbrand

**Affiliations:** ^1^Institute of Molecular Pathobiochemistry, Experimental Gene Therapy and Clinical Chemistry, RWTH University Hospital AachenAachen, Germany; ^2^Department of Internal Medicine I, University Hospital RegensburgRegensburg, Germany

**Keywords:** xanthohumol, hops, fibrosis, hepatic stellate cells, liver disease

## Abstract

Xanthohumol is the principal prenylated flavonoid of the female inflorescences of the hop plant. In recent years, various beneficial xanthohumol effects including anti-inflammatory, antioxidant, hypoglycemic activities, and anticancer effects have been revealed. This review summarizes present studies indicating that xanthohumol also inhibits several critical pathophysiological steps during the development and course of chronic liver disease, including the activation and pro-fibrogenic genotype of hepatic stellate cells. Also the various mechanism of action and molecular targets of the beneficial xanthohumol effects will be described. Furthermore, the potential use of xanthohumol or a xanthohumol-enriched hop extract as therapeutic agent to combat the progression of chronic liver disease will be discussed. It is notable that in addition to its hepatoprotective effects, xanthohumol also holds promise as a therapeutic agent for treating obesity, dysregulation of glucose metabolism and other components of the metabolic syndrome including hepatic steatosis. Thus, therapeutic xanthohumol application appears as a promising strategy, particularly in obese patients, to inhibit the development as well as the progression of non-alcoholic fatty liver disease.

## Introduction

**Figure 1:**
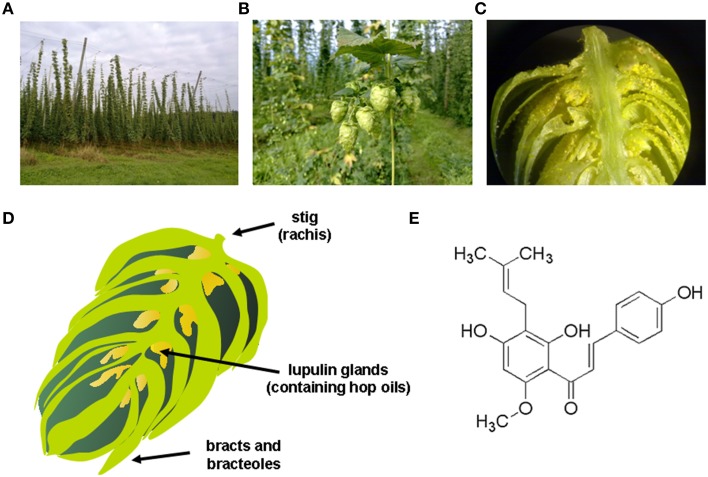
**Hop plant and xanthohumol. (A)** Hop plant (*Humulus lupulus*) field in Bavaria, showing the typical form of cultivation on high trolleys. **(B)** Female hop flowers (hop cones), where the biologically active substances, which are also important for brewing, are concentrated inside in the lupulin glands; **(C)** lupulin glands, which contain hop resins, bitter acids, essential oils and prenylated flavonoids. **(D)** Schematic drawing of a female hop cone that is composed of a central spine (i.e., the strig), bracts (i.e., pear-shaped petal that does not contain lupulin glands), bracteoles (i.e., pear-shaped petal of the hop cone that shelters the lupulin glands and any seeds that may be present), and the characteristic lupulin glands that are tiny yellow sacs containing the hop oils. **(E)** Chemical structure of xanthohumol (3′-[3,3-dimethyl allyl]-2′,4′,4-trihydroxy-6′-methoxychalcone), the major prenylated chalcone of the hop plant.

Hop (*Humulus lupulus* L.) has been used since ancient times as a medicinal plant. Traditional medicinal indications included the treatment of anxiety and insomnia, mild pain reduction or combating dyspepsia (Zanoli and Zavatti, 2008). Today, hops are used in the manufacturing of beer and female infertile plants are cultivated on high trolleys especially for brewing (Figure 1A). Biologically active substances, which are also important for brewing, are concentrated inside hop cones (Figure 1B) in lupulin glands (Figure 1C) which contain hop resins, bitter acids, essential oils and prenylated flavonoids. These lupulin glands are tiny yellow sacs that are located at the base of the petals of the hop cone (Figure 1D) that are found in female plants, while cones from the male hop plant contain relatively few lupulin glands.

Xanthohumol (XN; 3′-[3,3-dimethyl allyl]-2′,4′,4-trihydroxy-6′-methoxychalcone) is the principal prenylated chalcone of the hop plant (Figure 1E). The yellow compound (Greek: xantho = yellow) is found in high quantities in the lupulin glands. Since the 1990s, interest in health-promoting activities of XN increased constantly, scientific investigations were initialized worldwide and papers and patents on this topic have increased steadily (Gerhauser and Frank, 2005).

**Figure 2:**
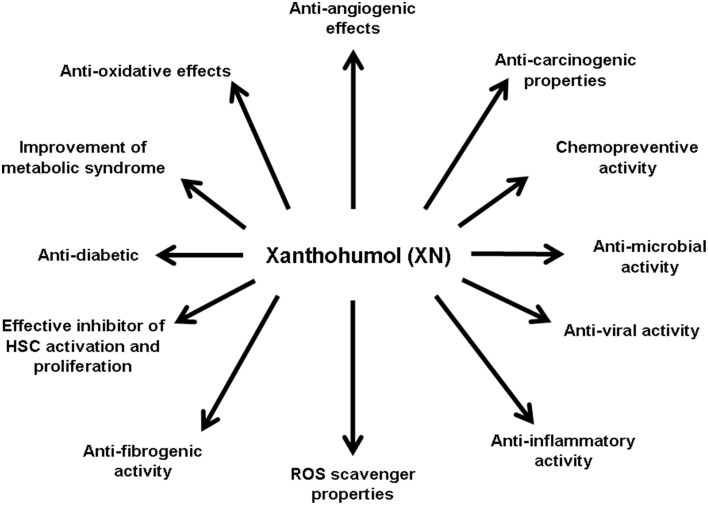
**Biological effects of xanthohumol**. Xanthohumol (XN) has been shown to have wide spectrum of biological effects, by which it may also affect different pathophysiological mechanisms involved in the development and progression of chronic liver disease. Studies have shown that XN acts anti-angiogenic (Albini et al., 2006; Shamoto et al., 2013), anti-carcinogenic (Dorn et al., 2010a,b,c; Araujo et al., 2011), chemopreventive (Miranda et al., 2000c; Gerhauser et al., 2002; Dorn et al., 2010a), anti-microbial (Gerhauser and Frank, 2005; Rozalski et al., 2013; Kramer et al., 2015), anti-viral (Buckwold et al., 2004; Zhang et al., 2009, 2010; Lou et al., 2014), anti-inflammatoric (Dorn et al., 2010a, 2013; Jongthawin et al., 2012), as a ROS scavenger (Gerhauser et al., 2002), anti-diabetic (Legette et al., 2013), and anti-oxidative (Gerhauser et al., 2002). In regard to liver fibrogenesis, it was shown that XN has anti-fibrogenic potential (Dorn et al., 2010a, 2013; Yang et al., 2013) and inhibits HSC activation and proliferation (Dorn et al., 2010a). Moreover, XN improves the metabolic syndrome (Legette et al., 2013).

Many studies identified XN as a broad-spectrum cancer chemopreventive agent acting by multiple mechanisms relevant for cancer development and progression (Gerhauser et al., 2002). XN is able to scavenge reactive oxygen species and it modulates many enzymes involved in carcinogen metabolism and detoxification (Gerhauser et al., 2002). Furthermore, XN inhibits cyclooxygenase 2 (COX-2) expression and the activity of both Cox-1 and Cox-2 in lipopolysaccharide-mediated iNOS induction in the macrophages (Stevens and Page, 2004). XN also has been shown to decrease prostaglandin-E2 (PGE2) expression (Jongthawin et al., 2012). These anti-inflammatory properties may contribute to the inhibition of tumor promotion by the inhibition of nuclear factor-κB (NF-κB) signaling and subsequent down-regulation of pro-inflammatory factors (Albini et al., 2006; Colgate et al., 2007). Moreover, estrogen-mediated tumor promotion may be prevented by XN, which suppresses estrogen-signaling through the inhibition of the interaction between the oncoprotein brefeldin A-inhibited guanine nucleotide-exchange protein 3 (BIG3) and tumor suppressor prohibitin 2 (PHB2) (Yoshimaru et al., 2014). XN also inhibits the enzyme aromatase (CYP19), which plays a crucial role in the conversion of testosterone to estrogen (Monteiro et al., 2006). Furthermore, XN inhibits tumor cell growth by different mechanism such as decrease of DNA polymerase alpha activity and inhibition of DNA synthesis (Gerhauser et al., 2002). Moreover, XN induces apoptosis by poly(ADP-ribose)polymerase (PARP) cleavage, activation of caspases or down-regulation of Bcl-2 protein expression (Pan et al., 2005). XN has further been shown to modulate drug metabolism *in vitro* by inhibition of various Cyp enzymes and by induction of quinone reductase activity (Henderson et al., 2000; Miranda et al., 2000a), which has been considered as a biomarker for cancer chemoprevention (Cuendet et al., 2006). In addition to the molecular mechanism by which XN affects cancer cells, it has been shown to exhibit several further biological effects (Figure 2) which are also playing an important role during the course of chronic liver disease. Hepatic fibrosis is the peril that determines morbidity and mortality in patients with liver disease. Cirrhosis, as the end stage of hepatic fibrosis, is a major clinical issue for its high prevalence in the world and its tight relationship with hepatocellular carcinoma (HCC) incidence (Gines et al., 2004; Villanueva et al., 2007; Minguez et al., 2009). The activation of hepatic stellate cells (HSC) is the key event of hepatic fibrosis (Lang and Brenner, 1999; Kisseleva and Brenner, 2007; Elpek, 2014). Activated HSC/myofibroblasts are the cellular source of the excessive extracellular matrix (ECM) deposition (Lang and Brenner, 1999; Kisseleva and Brenner, 2007). Furthermore, activated HSC/myofibroblasts form and infiltrate the tumor stroma and promote HCC progression (Amann et al., 2009). Therefore, these cells are a critical target for therapy during the whole course of chronic liver disease. However, up to date, no effective therapy is available to block the activation of HSC or to inhibit the pro-inflammatory and pro-fibrogenic activity of the activated HSC. In the following, we provide a summary of present studies indicating the potential of this hop constituent as a therapeutic agent to beneficially affect hepatic fibrosis as well as various further pathological mechanisms during the course of chronic liver disease.

## Effects of xanthohumol on hepatic stellate cells *in vitro*

**Figure 3:**
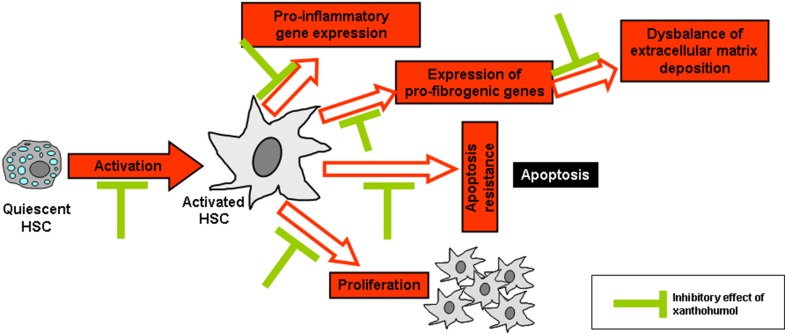
**Reported *in vitro* effects of xanthohumol in hepatic stellate cells**. Xanthohumol (XN) inhibits the activation of hepatic stellate cells (HSC) as well as several key pathological factors of activated HSC including pro-inflammatory and -fibrogenic gene expression, proliferation, apoptosis resistance, and composition of extracellular matrix. In particular, it is known that XN inhibits pro-inflammatory and pro-fibrogenic (Dorn et al., 2010a, 2013) gene expression and HSC activation (Yang et al., 2013) thereby preventing the excessive formation and deposition of extracellular matrix as well as the activation and proliferation of HSC. In addition, XN was shown to interfere with HSC apoptosis (Dorn et al., 2010a).

As already mentioned, the activation of HSC plays a critical pathophysiological role in the progression of chronic liver disease and the activation of these cells in response to liver injury is considered as the key event of hepatic fibrosis (Bataller and Brenner, 2001). Interestingly, XN has been shown to inhibit the activation of primary human HSC *in vitro* in concentrations as low as 5 μM XN (Dorn et al., 2010a). Furthermore, XN induced apoptosis in activated HSC *in vitro* in a dose-dependent manner (0–20 μM). Moreover, XN reduced expression of the pro-inflammatory factors such as the monocyte chemoattractant protein-1 (MCP-1) or pro-fibrogenic genes such as type I collagen in HSC. MCP-1 is regulated by NF-κB and increased levels are associated with fibrosis progression in chronic liver disease (Jarrar et al., 2008; Wouters et al., 2008). Further, NF-κB activation is a central pathophysiological mechanism during HSC activation (Hellerbrand et al., 1998a,b; Elsharkawy et al., 2005). Importantly, XN inhibited NF-κB activity in activated HSC *in vitro* (Dorn et al., 2010a). In summary, *in vitro* studies revealed that XN inhibits several key pathological factors of HSC activation and their contribution to fibrosis progression in chronic liver disease (Figure 3). In addition, XN has been shown to inhibit HSC-activation and hepatic fibrosis in experimental models of liver injury (please see below). Future research, may aim at the identification of further molecular pathways which may contribute to the anti-fibrogenic effect of XN in HSC.

## Anti-viral and antimicrobial activities of XN

HSC activation occurs in response to hepatocellular injury, and hepatitis C virus (HCV) infection is a one of the major causes of liver infectious diseases. *In vitro* studies using virus that causes bovine diarrhea (bovine viral diarrhea virus - BVDV E2), which shows considerable similarities with the human HCV, showed that XN inhibits BVDV replication and enhanced the anti-viral activity of interferon (IFN)-α (Buckwold et al., 2004; Zhang et al., 2009, 2010). Antiviral activity of XN in combination with IFNα-2b, was also demonstrated against herpes simplex virus and cytomegalovirus (Buckwold et al., 2004). More recently, XN was examined for its ability to inhibit HCV virus replication in a cell culture system carrying replicating HCV RNA replicon and it was shown that XN has similar inhibitory effects as IFNα-2b (Lou et al., 2014). Bacteria and bacterial translocation from the intestine have been shown to promote the progression of chronic liver disease, including HSC-activation and fibrogenesis (Seki et al., 2007). More recently, it has been shown that antibiotics improved the intestinal permeability and attenuated liver fibrosis development associated with Nonalcoholic Steatohepatitis (NASH) *via* the inhibition of HSC activation (Douhara et al., 2015). With regards to this, it is important, that the broad spectrum of antimicrobial activity of XN is well known (Gerhauser, 2005). More recently, the inhibitory effect of a hop extract containing XN was demonstrated against different gram-positive bacteria including *Listeria monocytogenes* and *Staphylococcus aureus* (Kramer et al., 2015).

In summary, there is a high rationale that XN may exhibit beneficial effects in liver disease also *via* inhibiting bacterial translocation or the growth of (gram-positive) bacteria. Still experimental proof of this hypothesis is missing, which may be the subject of future research in appropriate (*in vivo*) models or clinical studies.

## XN effects on hepatocellular cancer cells and primary hepatocytes

As already mentioned, a number of studies indicated the potential of XN to prevent and treat different types of cancers (Araujo et al., 2011). In the pathogenesis of HCC, XN exhibited anti-tumorous effects and induced the apoptosis in two different human HCC cell lines (HepG2 and Huh7) *in vitro* at concentration of 25 μM (Dorn et al., 2010b). Furthermore, XN repressed proliferation and migration in both cell lines even at lower concentrations (Dorn et al., 2010a). In a study with HepG2 cells, anti-mutagenic effects of XN were demonstrated even at concentrations of 0.01–0.1 μM (Plazar et al., 2008; Viegas et al., 2012). Chemopreventative effects can also occur by detoxifying carcinogens through the action of specific enzymes. One of these enzymes is NAD(P)H: quinone reductase that catalyzes the reduction of quinone to hydroquinones, which are more suitable substrates for subsequent conjugation. It was found that XN increased by several fold the activity of quinone reductase in murine liver cells (Hepa-1c1c7) (Miranda et al., 2000b) at concentrations above 1 μM. Importantly, even at XN concentrations as high as 100 μM, XN did not affect the viability of primary human hepatocytes *in vitro* (Dorn et al., 2010a).

It should be noted that in cellular experiments performed with XN the reproducibility of all these findings might be affected by the low solubility of XN in cell culture medium, the composition of media used, the tendency of XN to absorb to various plastic materials routinely used in the cell culture, and the reduction of the effective XN dose by conversion to isoxanthohumol (Motyl et al., 2012). Likewise, the bioavailability of XN and its prenylated flavanone (i.e., Isoxanthohumol) is marked by inter-individual variability that is induced by variations in the intestinal microbial community and their degradation pathways that have direct impact on biotransformation of respective compounds (Possemiers et al., 2005).

Therefore, studies that investigate pharmacological effects of XN or its natural or synthetic derivatives should punctiliously describe all details of the chosen experimental setting, not only including sources and concentrations of employed prenylflavonoid but also specification of media and buffer composition, identity and origin of cell lines and animals, details about culture conditions, animal accommodation, supplier of plastic ware, and many others.

Furthermore, future research may focus on the identification of (downstream) signaling pathways responsible for xanthohumol effects in hepatocytes. Recently, it has been shown that the activation of Nrf2 pathway and subsequently phase II enzymes in concert with p53 induction may account for the molecular mechanism of the chemopreventive activity of XN in hepatocytes (Krajka-Kuzniak et al., 2013). On the other hand its cytotoxicity toward HCC cells indicates that it may also be considered as potentially chemotherapeutic (Dorn et al., 2010a). Moreover, the antimutagenic effects of XN were demonstrated against various procarcinogens, which are activated by cytochrome P450 enzymes (Plazar et al., 2007; Ferk et al., 2010). The mechanisms are possibly related to the inhibition of the metabolic activation by human cytochrome P450 1A2 (CYP1A2) and inhibition of the binding of the metabolites to DNA and proteins (Miranda et al., 2000c). Moreover, XN interferes with several stages in the angiogenic process, including inhibition of endothelial cell invasion and migration, growth, and formation of tubular-like structures (Gerhauser et al., 2002; Albini et al., 2006). The mechanisms for its inhibition of angiogenesis are related to the blockage of both the nuclear factor-κB (NFκB) and Akt pathways in endothelial cells (Albini et al., 2006). Furthermore, in addition to the direct effect of XN on the vascular cells, XN inhibits the production of angiogenic factors, e.g., vascular endothelial growth factor (VEGF) and interleukin 8 (IL-8) via the inhibition of NF-κB (Shamoto et al., 2013). Still, according analyses in primary hepatocytes are missing.

## XN effects in models of acute liver injury

There is already a multitude of reports describing beneficial effects of XN on liver injury *in vitro* and in *vivo* (Table 1). Hepatic ischemia/reperfusion (I/R) injury occurs in a variety of clinical scenarios, including transplantation, liver resection, trauma, and hypovolemic shock. The process of hepatic I/R-injury can be divided into two phases; an acute phase (the first 6 h after reperfusion) and the following sub-acute phase (Fan et al., 1999). The acute phase is characterized by generation of reactive oxygen species (ROS) subsequent to reoxygenation of the liver leading to marked hepatocellular damage (Parks and Granger, 1988; Rauen et al., 1994). Noteworthy, pretreatment of mice with XN (1,000 mg/kg body weight for 5 days) significantly ameliorated I/R-induced oxidative stress 6 h after reperfusion (Dorn et al., 2013). Although hepatocellular damage was not modulated at this early phase, the I/R-induced NF-κB activation and pro-inflammatory gene expression was almost completely blunted (Dorn et al., 2013). These factors play a crucial role in the later course of hepatic I/R-injury *via* recruitment and activation of pro-inflammatory cells (Jaeschke et al., 1992; Jaeschke, 1996). Also in an *ex vivo*-model of cold hepatic I/R XN revealed an antioxidant and inhibitory effect on NF-κB activity (Hartkorn et al., 2009).

**Table 1:** **Beneficial effects of Xanthohumol on liver injury**.

**Finding made in**	**Model**	**Biological activity**	**References**
*In vitro* (cell culture model)	Hepatitis C virus replication in cell culture	XN reduced hepatitis C virus RNA levels	Lou et al., 2014
	Cultivated human hepatocytes and hepatic stellate cells	In both cell types XN inhibited activation of the transcription factor NF-κ B and expression of NF-κ B dependent proinflammatory genes	Dorn et al., 2010a
	Hepatocellular carcinoma cell lines (HepG2 and Huh7)	XN induced apoptosis and repressed proliferation and migration as well as TNF-induced NF-κ B activity	Dorn et al., 2010b
	Comet assay in cultured human hepatoma cell line HepG2	XN prevents the formation of DNA strand breaks, indicating that its protective effect is mediated by induction of cellular defense mechanisms against oxidative stress	Plazar et al., 2007
*In vitro* (Precision cut liver slices)	Precision-cut rat liver slices	XN completely prevented 2-amino-3-methylimidazo[4,5-f]quinoline- and benzo(a)pyrene-induced DNA damage	Plazar et al., 2008
*In vivo* (mice)	Ischemia-reperfusion (I/R) induced liver injury in BALB/c mice	Orally applied XN (1 mg/g body weight for 5 days before I/R-injury) reduced liver injury, NF-κ B activation, expression of proinflammatory cytokines	Rauen et al., 1994
	Carbon tetrachloride-induced acute liver injury in 10 weeks old female BALB/c mice	XN inhibited pro-inflammatory and profibrogenic hepatic gene expression and decreased hepatic NF-κ B activity	Dorn et al., 2012
	Western-type diet-fed ApoE-deficient mice	XN reduced plasma cholesterol concentrations, decreased atherosclerotic lesion area, and attenuated plasma concentrations of the proinflammatory cytokine monocyte chemoattractant protein 1	Doddapattar et al., 2013
	Mouse model of Non-Alcoholic Steatosis	XN reduced hepatic inflammation and expression of profibrogenic genes	Dorn et al., 2010a
*In vivo* (Tupaia)	Hepatitis C virus infected *Tupaia belangeri*	XN improves hepatic inflammation, steatosis and fibrosis through inhibition of oxidative reaction and regulation of apoptosis and suppression of hepatic stellate cell activation	Yang et al., 2013
*In vivo* (rat)	High fat diet in rats	XN inhibited the increase of body weight, liver weight, and triacylglycerol levels	Yui et al., 2014
	Orally administered hop extract and subcutaneously injection of XN in Sprague-Dawley rats over 4 days	XN display cytoprotective effects in the liver	Dietz et al., 2013
	Carbon tetrachloride-induced acute liver injury in rats	XN evolves hepatoprotective effects by its antioxidant properties and inhibition of lipid peroxidation and degradation of antioxidant enzymes that are induced by CCl_4_ intoxication	Pinto et al., 2012
	Metabolic syndrome in 4 week old Zucker fa/fa rats	XN has beneficial effects on markers of metabolic syndrome such as body weight and plasma glucose levels	Legette et al., 2013
	Amino-3-methyl-imidazo[4,5-*f*]quinoline-induced preneoplastic foci formation in rat livers	XN protects against DNA damage and cancer	Pinto et al., 2012
	IR-induced hepatic injury in rats	XN reduced reactive oxygen species formation and NF-κ B activity *in vitro* and lipid peroxidation was attenuated, while Bcl-X expression and caspase-3 like activity was decreased	Hartkorn et al., 2009

*Abbreviations used are: I/R, Ischemia-reperfusion; NF-κB, nuclear factor-κB; XN, Xanthohumol*.

The liver is also frequently exposed to various insults, including toxic chemicals (Zimmerman and Lewis, 1995; Grunhage et al., 2003). Liver damage caused by hepatotoxic chemicals induces liver necrosis due to direct damage of hepatocytes and subsequent inflammation (Mehendale et al., 1994). Carbon tetrachloride (CCl_4_), an industrial solvent, is a hepatotoxic agent and its administration is widely used as an animal model of toxin-induced liver injury that allows the evaluation of both necrosis and subsequent inflammation (Huh et al., 2004) as well as fibrosis (Iredale, 2007). In this model, oral application of XN (500 mg/kg BW) was shown to significantly inhibit the pro-inflammatory and pro-fibrogenic hepatic gene expression (Dorn et al., 2012). Noteworthy, these effects occurred despite the fact that hepatocellular injury as reflected by serum levels of transaminases or histomorphological analysis was comparable between control mice and XN-fed mice. These findings suggest that the suppressive effect of XN against the progress of acute CCl_4_-induced hepatic fibrosis involved direct mechanisms related to its ability to block both hepatic inflammation and the activation of HSC.

In summary, these findings indicate the potential of XN to exhibit a beneficial effect in acute liver injury or failure. Still, it has to be mentioned that in the studies cited above XN has be applied before the onset of liver injury, i.e., prevented acute liver injury in experimental models. Further studies are warranted to analyzed the therapeutic potential of XN, i.e., after the onset of liver injury. A further potential clinical application of XN might be the prevention of oxidative stress in conservation solutions during the organ transplantation process. However, also here, future studies are warranted.

## Effect of XN in models of chronic liver injury

Chronic HCV infection is one of the most frequent liver diseases worldwide (Yang et al., 2013). In an elegant study, Yang et al. analyzed the hepatoprotective effect of XN in an *in vivo* model of HCV infected *Tupaias*. XN was applied by gavage at a dose of 100 mg/kg BW which led to a significant reduction of hepatic inflammation and fibrosis compared to control animals. Interestingly, XN also inhibited the hepatic steatosis in this model, which was found to be related to an inhibitory effect on microsomal triglyceride transfer protein activity and inhibition of hematopoietic stem cells (Yang et al., 2013).

Besides alcohol abuse, non-alcoholic fatty liver disease (NAFLD) has emerged as the most frequent liver disease in Western countries (Clark et al., 2002; Cobbold et al., 2010; Vernon et al., 2011). Today, the metabolic syndrome (MS) is one of the major public health challenges worldwide that is characterized by clustering of waist circumference, blood triglycerides, high density lipoprotein (HDL) cholesterol and fasting glucose levels. MS is also closely associated with NAFLD, and thus today, NAFLD is considered as a component of the MS (reviewed in Saidijam et al., 2014). MS affects approximately 25 per cent of the adult population in Western countries and also is quickly increasing in young populations. Accordingly, also NAFLD incidence is further increasing and effective strategies to prevent the development and progression of NAFLD to its advanced form NASH are urgently needed. Of note, XN has been shown to exhibit a beneficial effect in different experimental NAFLD models. Yui et al. reported that feeding rats a high-fat diet enriched with XN extract (1% w/w equivalent to a dose of 100 mg/kg BW) inhibited the increase of body and liver weight, as well as triacylglycerol levels in the plasma and in the liver (Yui et al., 2014). The mechanisms were found to be related to the regulation of the hepatic fatty acid metabolism and an inhibition of fat absorption in the intestine (Doddapattar et al., 2013). In this study, XN also tended to reduce hepatic fatty acid synthesis through the reduction of hepatic sterol regulatory element-binding protein (SREBP) 1c mRNA expression in rats fed with a high-fat diet. Furthermore, it was observed that plasma adiponectin levels tended to be elevated by dietary application of the XN-rich hop extract (Yui et al., 2014)^.^ Also in a second model, in which NASH was induced by feeding the mice with an NASH-inducing diet, XN exhibited anti-inflammatory and anti-fibrogenic effects (Dorn et al., 2010a). Here, XN was applied in the diet in a concentration of 1% W/W corresponding to a dose of approximately 1000 mg/kg BW. In this model, after 3 weeks feeding, the induction of hepatic inflammation and pro-fibrogenic gene expression was almost completely blunted in mice receiving XN-supplemented NASH diet compared to mice fed with the pure NASH-diet (Dorn et al., 2010a).

Moreover, ApoE^−/−^ mice showed decreased hepatic triglyceride and cholesterol content, activation of AMP-activated protein kinase, phosphorylation and inactivation of acetyl-CoA carboxylase, and reduced expression levels of mature SREBP-1c upon 8 weeks XN feeding (300 mg/kg BW/day) (Doddapattar et al., 2013).

In summary, XN has been shown to beneficially affect several components of the metabolic syndrome. Furthermore, it positively affects other obesity associated pathological factors such as misbalance of adipokine levels, which are known to promote NAFLD development and progression. Fitting to this is the beneficial effect of XN on hepatic steatosis, inflammation and fibrosis in experimental NASH models. In addition to NAFLD/NASH, XN revealed positive effects in other experimental models of chronic liver injury such as HCV, which can be explained by the above described inhibitory effects on fibrogenic and inflammatory gene expression or anti-bacterial effects. Still, it has to be noted that in the published studies, XN was applied in a preventive experimental setting. Future studies are warranted to analyze the potential of XN to treat, i.e., stop or reverse liver fibrosis.

## Pharmacokinetics and effective dose of XN

Recent studies enhanced our knowledge regarding metabolisms and pharmacokinetics of XN (Nookandeh et al., 2004; Legette et al., 2012, 2014). Investigations using human liver microsomes showed that the hydroxylation of a prenyl methyl group is the primary route of the oxidative metabolism. Furthermore, XN and its metabolites were found to be excreted mainly in feces within 24 h of administration, when XN was fed to rats up to a dose of 500 mg/kg BW (Avula et al., 2004; Hanske et al., 2010). Twenty two metabolites were identified in the feces, most of them confined to modified chalcone structures and flavanone derivatives (Nookandeh et al., 2004). Still, most of the XN remained unchanged in the intestinal tract (89%), and only 11% were found to be metabolites (Nookandeh et al., 2004). Furthermore, two phase I metabolites and five phase II metabolites were identified in rats revealing oxidation, demethylation, hydration and sulfatation reactions (Jirasko et al., 2010). The bioavailability of XN in rats was found to be dose-dependent (0.33, 0.13, and 0.11) upon oral administration of single XN doses (1.86, 5.64, and 16.9 mg/kg BW) (Legette et al., 2012).

Despite the poor bioavailability, the highest XN concentrations are reached in intestinal cells and non-parenchymal liver cells upon oral uptake. Here, the anatomical situation of the liver has to be considered. Thus, it can be expected, that after oral intake of XN its concentration in the portal vein is higher than in the systemic circulation. Further, HSC are located in the liver in the space of Disse (perisinusoidal space), i.e., between the sinusoids and the hepatocytes. Herewith, HSC are directly exposed to XN concentration reaching the liver *via* the portal vein irrespective of the (subsequent) metabolism in hepatocytes. Anti-fibrogenic effects have been observed at concentrations as low as 5 μM (Dorn et al., 2010a), and in previous studies we found that these concentration levels are reached in the murine hepatic tissue upon oral administration of XN at dose of 1000 mg/kg BW (Dorn et al., 2013). Furthermore, it has to be considered that XN concentrations reaching HSC in the space between the endothelial cells and the hepatocytes (i.e., space of Disse) likely are significantly higher than the levels in whole liver tissue.

Still, the applied dose of 1000 mg/kg BW in this study (Dorn et al., 2013) was quite high and it has not yet been exactly defined what XN doses are required to achieve hepatoprotective effects. In the above described studies revealing beneficial effects in models of acute and chronic liver injury, XN was applied in the dose-range of approximately 100–1000 mg XN/kg BW to mice and rats. Effective doses with regards to beneficial effects on other components of the metabolic syndrome were achieved with doses in the range of 15–300 mg XN /kg BW in rats and mice (Doddapattar et al., 2013; Legette et al., 2013; Yang et al., 2013; Yui et al., 2014). In addition, there are several further studies in other experimental disease models, which revealed beneficial effects in doses as low as 0.2–9 mg XN/kg BW (Benelli et al., 2012; Negrao et al., 2012; Rudzitis-Auth et al., 2012; Yen et al., 2012; Costa et al., 2013). For example, Costa et al. have shown that XN modulates inflammation, oxidative stress, and angiogenesis in a type 1 diabetic rat skin wound healing model by supplementing a stout beer with 10 mg XN/L, which corresponds to approximately 1 mg XN/kg BW/day (Costa et al., 2013). Moreover, Benelli et al. reported that XN revealed beneficial effects in a murine model of leukemia in doses as low as 2 mg XN/kg BW (Benelli et al., 2012).

## Potential forms of XN application

Elegant studies by Legette et al. (2012) demonstrated the similarity of XN metabolisms and pharmacokinetics between rodents and humans, which allows the translation of data generated in murine and rat models into clinical studies/application. To convert an animal dose (mg/kg BW) to the human equivalent dose (HED), the murine dose should be either divided by 12.3 or multiplied by 0.08. Oral dose ratio can be calculated using allometric interspecies scaling ^1^.

For humans, beer is the major dietary source of XN. The beer content of XN varies significantly depending on the type of beer (in the range of 0.052–0.628 mg/l) (Chen et al., 2010). Lager and Pilsener beers have fairly low levels of this compound, and the highest levels of XN are found in Stout or Porte (Stevens et al., 1999). Moreover, it was possible to produce a dark beer enriched in XN (3.5 mg/l) (Magalhães et al., 2008), and a brewing process has been developed that produces a beer that contains 10 times the amount of XN as traditional brews (Wunderlich et al., 2005). Even upon consumption of such special types of beer, a daily beer consumption of approximately 150–1500 l would be necessary by a man (75 kg) to reach doses corresponding to 100–1000 mg XN/kg BW/day in mice, i.e., doses which have been shown to exhibit hepatoprotective effects. Thus, with regards to hepatoprotective effects, pharmacologically relevant XN concentrations cannot be reached in men by beer consumption. Moreover, there is certainly unanimous hesitancy among researchers to recommend drinking alcohol to avoid any kind of disease because of the fine line between moderate and binge drinking. Certainly, this is even more true in case of chronic liver disease.

However, XN can also be isolated from hops in large quantities, and different methods (e.g., extraction via liquid supercritical carbon dioxide) were developed to isolate XN from hop cones in large quantities. Thus, independent of beer intake, XN may be used as hepatoprotective dietary supplement. Therefore, pharmacological relevant concentrations can be reached by oral administration of XN enriched functional food, e.g., XN enriched beverages or solid foods. Methods or formulations for increasing the water solubility and bioavailability are presently object of numerous national or global patents^2^).

## Safety of XN application

One prerequisite for therapeutic application is the good safety profile of the used agent. Especially hepatotoxic properties have to be excluded, when the therapeutic agent must be taken by patients with liver disease. Hop has a long history as a medicinal plant and is known for its good tolerance. More recently, we and others have confirmed the safety of oral application of XN and XN-enriched hop extracts in rats and mice. Oral administration of XN (700 mg/kg/day BW) to mice for 4 weeks did neither affect the major organ functions, nor the protein, lipid, or carbohydrate metabolism (Vanhoecke et al., 2005). Similarly, mice fed with a XN enriched diet (1000 mg/kg BW) for 3 weeks exhibited no adverse effects (Dorn et al., 2010a). Histopathological evaluation of major organs (liver, kidney, colon, lung, heart, spleen, and thymus) as well as biochemical serum analysis, confirmed that XN did not negatively affect organ function and homoeostasis (Dorn et al., 2010c). Most recently, first in man studies confirmed the safety and good tolerability of XN or XN-enriched hop extracts, respectively. An escalating dose study (up to 1.35 mg XN/kg BW/day for 1 week) in menopausal women revealed that the XN enriched extract did neither affect the serum levels of sex hormones nor blood clotting. Also no other side effects were observed (van Breemen et al., 2014). A further study confirmed the safety of oral administration of a single XN dose (160 mg, i.e., approximately 2.5 mg XN/kg BW/day) in healthy volunteers (Legette et al., 2014).

## Summary and conclusion

**Figure 4:**
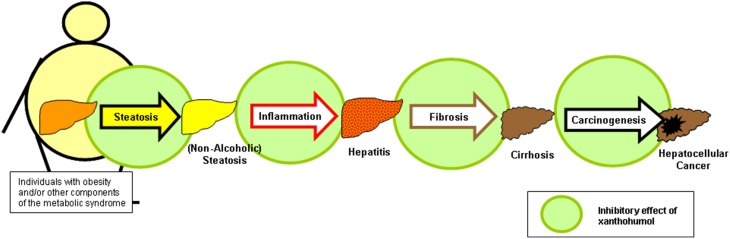
**Xanthohumol effects on different pathophysiological factors during the course of non-alcoholic fatty liver disease**. Xanthohumol (XN) has the potential to inhibit hepatic steatosis, inflammation, fibrosis and even the causes of liver injury by interaction on different levels, i.e., pathogenesis of steatosis (Dorn et al., 2010a; Doddapattar et al., 2013; Yang et al., 2013; Yui et al., 2014), inflammation (Dorn et al., 2010a, 2013; Yang et al., 2013), fibrosis (Dorn et al., 2010a, 2013; Yang et al., 2013), and cancerogenesis (Gerhauser et al., 2002; Dorn et al., 2010b).

*In vitro* and *in vivo* data indicate that the hop constituent xanthohumol (XN) affects several critical pathophysiological steps during the development and course of chronic liver disease, including hepatic inflammation and fibrosis, as well as the formation and progression of liver cancer (Figure 4). Also on the molecular level, XN ameliorates several mechanisms which play a critical role in the pathogenesis of acute and chronic liver injury. Strikingly, inhibitory XN effects on activated hepatic stellate cells (HSC)/myofibrobasts take place in a concentration range, which is significantly lower than the concentration which is shown to be safe for primary human hepatocytes *in vitro*. Furthermore, upon oral application, HSC are exposed to relatively high XN concentrations due to their location in the space of Disse irrespective of the hepatic metabolisms. This indicates these non-parenchymal liver cells as attractive targets for therapeutic XN application. Of note, XN also holds promise as a therapeutic agent for treating obesity, dysregulation of glucose metabolism and other components of the metabolic syndrome including hepatic steatosis. Thus, therapeutic XN application appears as promising strategy, particularly in obese patients, to inhibit the development as well as the progression of NAFLD.

### Conflict of interest statement

Part of the xanthohumol-related research projects from Claus Hellerbrand are funded by Flaxan, a subsidiary of Joh. Barth and Sohn GmbH (Nuremberg, Germany), and by “Wissenschaftsförderung der Deutschen Brauwirtschaft e.V.” (Berlin, Germany). Claus Hellerbrand is a consultant for Flaxan, and Abdo Mahli is working in the laboratory of Claus Hellerbrand. Ralf Weiskirchen and Sabine Weiskirchen have nothing to declare.

## Footnotes

^1^Guidance for Industry, Estimating the maximum safe starting dose in initial clinical trials for therapeutics in adult healthy volunteers. This guidance can be downloaded at http://www.fda.gov/downloads/Drugs/GuidanceComplianceRegulatoryInformation/Guidances/UCM078932.pdf (last downloaded March 20, 2015).

^2^International application published under the patent cooperation treaty (PCT). A representative example can be downloaded at http://www.lens.org/images/patent/WO/2007016578/A2/WO_2007_016578_A2.pdf (last downloaded March 20, 2015).

## Abbreviations

BW: body weight
ECM: extracellular matrix
HCC: hepatocellular carcinoma
HCV: hepatitis C virus
HSC: hepatic stellate cells
IFN: interferon
MS: metabolic syndrome
NF-κB: nuclear factor-κB
XN: xanthohumol.
